# HIV-1 Replication and the Cellular Eukaryotic Translation Apparatus

**DOI:** 10.3390/v7010199

**Published:** 2015-01-19

**Authors:** Santiago Guerrero, Julien Batisse, Camille Libre, Serena Bernacchi, Roland Marquet, Jean-Christophe Paillart

**Affiliations:** Architecture et Réactivité de l’ARN, CNRS, Université de Strasbourg, Institut de Biologie Moléculaire et Cellulaire, 15 rue René Descartes, 67084 Strasbourg cedex, France; E-Mails: santiago.guerrero@crg.eu (S.G.); j.batisse@ibmc-cnrs.unistra.fr (J.B.); c.libre@ibmc-cnrs.unistra.fr (C.L.); s.bernacchi@ibmc-cnrs.unistra.fr (S.B.); r.marquet@ibmc-cnrs.unistra.fr (R.M.)

**Keywords:** HIV-1, translation, auxiliary proteins, IRES, leaky-scanning, frameshift, ribosome shunting

## Abstract

Eukaryotic translation is a complex process composed of three main steps: initiation, elongation, and termination. During infections by RNA- and DNA-viruses, the eukaryotic translation machinery is used to assure optimal viral protein synthesis. Human immunodeficiency virus type I (HIV-1) uses several non-canonical pathways to translate its own proteins, such as leaky scanning, frameshifting, shunt, and cap-independent mechanisms. Moreover, HIV-1 modulates the host translation machinery by targeting key translation factors and overcomes different cellular obstacles that affect protein translation. In this review, we describe how HIV-1 proteins target several components of the eukaryotic translation machinery, which consequently improves viral translation and replication.

## 1. Introduction

Eukaryotic translation is a complex process orchestrated by a wide range of players, including several protein factors and three classes of RNA (ribosomal RNA (rRNA), transfer RNA (tRNA), and messenger RNA (mRNA)). This process is comprised of three main steps: initiation, elongation and termination. Immediately after transcription, mRNAs are maturated and exported through the nuclear pores to the cytoplasm. Once in the cytoplasm, cap-dependent translation is initiated by the recruitment of the small ribosomal subunit (40S), which scans along the mRNA until it finds an initiation codon. At this point, the large ribosomal subunit (60S) is engaged to decode the mRNA and assembles amino acids to synthesize proteins. The elongation step is completed when the ribosome reaches a stop codon that triggers the termination step. Finally, the components of the translational machinery are recycled for further protein translation.

Optimal viral protein synthesis often occurs at the expense of cellular proteins and many viruses have evolved mechanisms that redirect and control the eukaryotic translational machinery. In these cases, viral factors can target the initiation, elongation and termination steps through interactions with key translation factors and mechanisms, which interfere with or disrupt the host translation machinery.

The HIV-1 proteins are mainly synthesized by a cap-dependent mechanism. Nonetheless, different pathways such as leaky scanning, frameshifting, ribosome shunting, and cap-independent mechanisms are used to complete translation of the viral proteome. Moreover, HIV-1 has evolved sophisticated strategies to overcome cellular barriers that affect viral protein translation. This is the case of ribosomal scanning inhibition due to highly structured RNA elements and cap-dependent translation inhibition triggered by host immune responses. In this review, we focus on the HIV-1 functions that are essential to control the cellular translation apparatus in order to improve translation of viral proteins at the expense of cellular factors.

## 2. Overview of Eukaryotic Translation

### 2.1 Translation Initiation

#### 2.1.1. Pre-Initiation Complex Assembly and mRNA Activation

**Figure 1:**
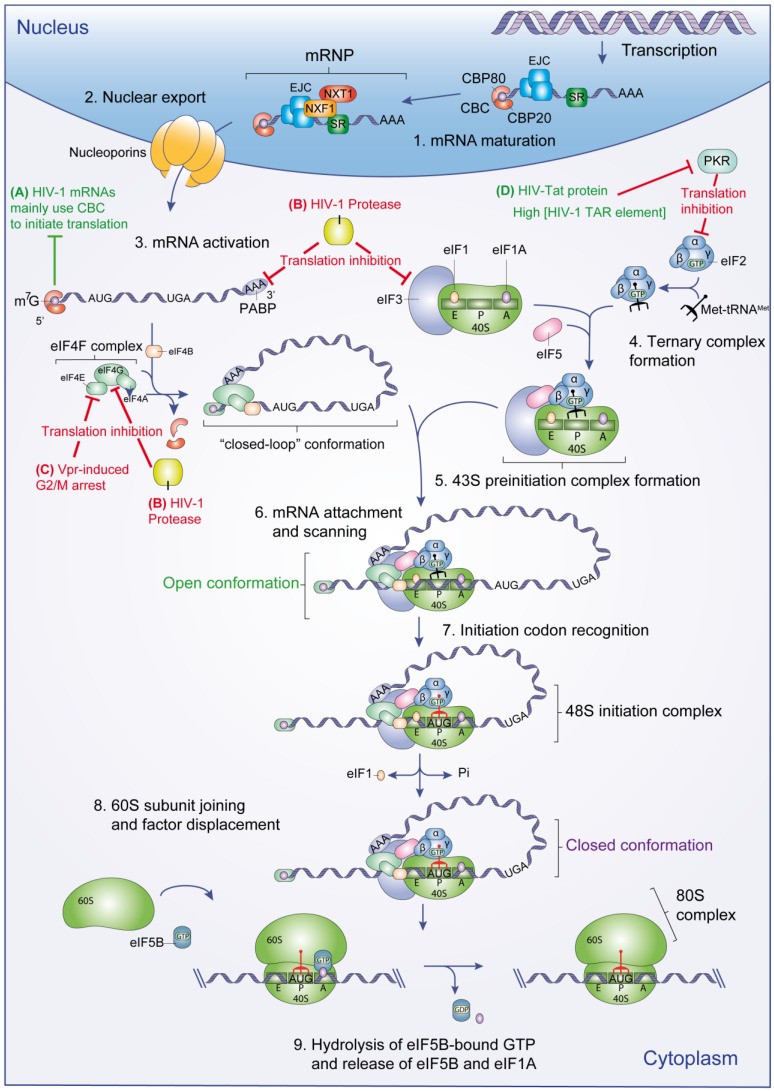
Eukaryotic translation initiation. mRNA maturation and nuclear export precede translation initiation (steps 1 and 2). Once in the cytoplasm, mRNA is activated (step 3) and 43S PIC formation takes place (step 4 to 5). The mRNA recruits the 43S PIC and scanning begins until an initiation codon is detected (step 6). At this point, the 48S initiation complex is formed and the 60S subunit is recruited to form the ribosome 80S complex (step 7 to 9). HIV-1 viral functions important to control the eukaryotic translation initiation are shown. (**A**) Unspliced and singly-spliced viral mRNAs could be translated due to the ability of the CBC to activate mRNA during translation initiation (green line); (**B**) HIV-1 protease partially inhibits translation initiation by targeting PABP, eIF3 and eIF4G (red lines); (**C**) Vpr-induced G2/M arrest indirectly inhibits host protein translation by targeting eIF4E activity (red line); (**D**) HIV-Tat protein and high concentration of HIV-1 TAR element indirectly promote viral translation by blocking PKR activity (red line). PKR phosphorylates eIF2α to block its recycling for ongoing translation, resulting in a potent translation inhibition of cellular and viral mRNA.

Prior to translation initiation, the 5'-end of nascent mRNAs are capped with a 7-methylguanosine (m^7^G) and subsequently polyadenylated at their 3'-end immediately after transcription. The m^7^G cap is then recognized by the cap-binding complex (CBC), which is composed by the cap-binding proteins 80 and 20 (CBP80/20) [1]. Messenger RNA export effectors (exon-exon junction complex (EJC) and SR proteins) are then loaded on mature mRNA molecules during the splicing events. These adaptors molecules allow the recruitment of the NXF1/NXT1 heterodimer, which mediates the export of the messenger ribonucleoprotein (mRNP) from the nucleus to the cytoplasm through the nuclear pores via interactions with nucleoporins [2,3] (Figure 1, steps 1–2).

Once in the cytoplasm, mRNAs undergo a CBC-mediated pioneer round of translation, which is important for the quality control of the transcript [4]. CBC is then displaced from the m^7^G and mRNAs are activated by the eukaryotic initiation factor 4F complex (composed of eIF4E, eIF4G and eIF4A) and eIF4B (Figure 1, step 3). Thus, mRNA acquires a “closed-loop” structure, required for an optimal mRNA recruitment into the 43S pre-initiation complex (PIC) (Figure 1, step 6). This conformation is achieved by the simultaneous binding of PABP (Poly(A)-binding protein) and eIF4E to eIF4G [5].

In parallel, ternary complex (TC) formation occurs through the assembly of the initiator methionyl-tRNA (Met-tRNA_i_^Met^), eIF2 and a GTP molecule (Figure 1, step 4). The TC is then recruited to the ribosomal subunit 40S to assemble the 43S PIC (Figure 1, step 5). In this process, other eIFs (eIF1, 1A, 3 and 5) are required to promote TC binding to the 40S subunit [6].

#### 2.1.2. Initiation Codon Recognition and 80S Complex Formation

Once the mRNA has been loaded on the 43S PIC, this complex scans the 5' untranslated region (5'UTR) until it recognizes a start codon (AUG) by complementarity with the anticodon of the Met-tRNA_i_. To prevent incorrect base pairing of the Met-tRNA_i_ to a non-AUG codon and to promote recognition of the correct start codon, 43S PIC employs a discriminatory sequence-based mechanism. This permissive sequence (GCC(A/G)CCAUGG) surrounding the start codon is termed Kozak consensus sequence and is principally composed of a purine at position −3 and a G at position +4 (the A of the AUG codon is designated as +1) [7,8,9]. Once the AUG start codon has been recognized and the 48S complex formation has been accomplished, eIF1 is ejected from the scanning complex (Figure 1, step 7) [10]. This in turn triggers hydrolysis of eIF2-bound GTP and Pi release by the eIF5 GTPase activity.

These events cause the transition from an “open” to a “closed” conformation of the scanning complex, which stabilizes the binding of the Met-tRNAi with the AUG start codon [11]. At this point, the remaining factors are dissociated to allow the joining of the 60S subunit and 80S complex formation (Figure 1, step 8). This process is mediated by eIF5B which causes the dissociation of eIF3, eIF4B, eIF4F and eIF5, and eIF5B self-dissociates from the assembled 80S ribosome by its GTPase activity [12]. This reaction also triggers the release of eIF1A to finally forms an elongation competent 80S ribosome (Figure 1, step 9) [13].

### 2.2. Translation Elongation and Termination

**Figure 2:**
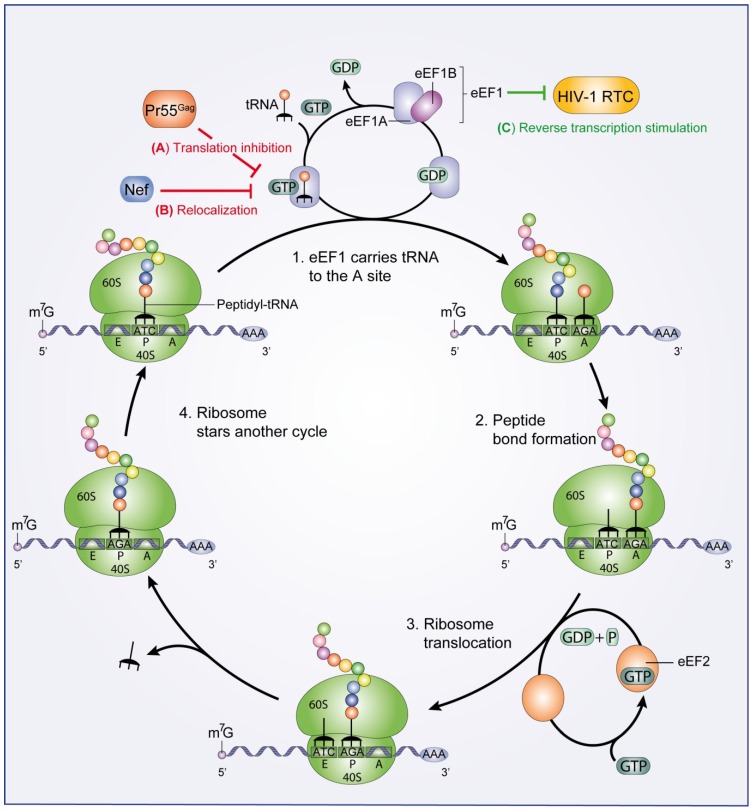
Eukaryotic translation elongation. eEF1A-GTP transports the aminoacylated tRNAs into the A site of the 80S ribosome and eEF1A-GDP is re-activated by eEF1B (step 1). The 80S ribosome-mediated peptide bond formation (step 2) precedes ribosome translocation mediated by eEF2 (step 3). Finally, the ribosome begins another cycle of peptide elongation (step 4). HIV-1 viral functions important to control the eukaryotic translation elongation are shown. (**A**) HIV-1 Pr55^Gag^ interacts with eEF1A and induces translation inhibition (red line); (**B**) HIV-1 Nef protein also interacts with eEF1A and mediates a nucleocytoplasmic relocalization of eEF1A (red line); (**C**) The HIV-1 RTC recruits eEF1 to stimulate late steps of the HIV-1 reverse transcription process (green line).

After the AUG start codon has been identified and the formation of the 80S ribosome has been achieved, translation elongation begins [13]. 80S ribosome decodes the mRNA sequence and mediates the addition of amino acids to elongate the growing polypeptide chain. Elongation is accomplished by the eukaryotic elongation factors eEF1 (composed of eEF1A and eEF1B) and eEF2 (Figure 2) [14,15]. The eEF1A-GTP complex carries each aminoacylated tRNA to the 80S ribosome A site where it builds codon-anticodon interactions (Figure 2, step 1). After 80S ribosome-catalyzed peptide bond formation (Figure 2, step 2), eEF2 mediates 80S ribosome translocation through GTP hydrolysis (Figure 2, step 3) and the next round of amino acid incorporation begins (Figure 2, step 4).

Polypeptide elongation continues until a stop codon triggers the translation termination step. This process is mediated by the eukaryotic translation termination factors eRF1 and eRF3 [16]. The eRF1 factor mediates stop codon recognition, while eRF3 potently stimulates peptide release. After protein release, eRF1 remains bound to the post-termination complex (post-TC), and in conjunction with the ATP-binding cassette protein ABCE1, dissociates the post-TC into the 60S subunit, and the tRNA- and mRNA-bound 40S subunit [17].

## 3. HIV-1 Takes Advantage of the Host Translation Machinery

### 3.1. Overcoming Ribosome Scanning Barriers

All spliced and unspliced HIV-1 transcripts possess the same 289 nt long 5'UTR. Because this region presents several highly structured motifs such as the trans-activation responsive (TAR) RNA element, the unwinding step of the ribosomal scanning process is expected to be inefficient [18]. Soto-Rifo* et al.* [19] have shown that RNA helicase DDX3 is required in order to overcome this constraint. DDX3 directly binds to the HIV-1 5'UTR and interacts with eIF4G and PABP to promote translation initiation of HIV-1 genomic RNA [19], and mediates pre-initiation complex assembly in an ATP-dependent manner. In this process, the HIV-1 genomic RNA seems to be located in large cytoplasmic RNA granules alongside with DDX3, eIF4G and PABP, but not with CBP20/80 and eIF4E [20].

*In vitro* experiments revealed that cytoplasmic DDX3 is able to bind the m^7^G cap independently of eIF4E, showing that DDX3 could promote the formation of a pre-initiation complex in the absence of eIF4E. Thus, DDX3 substitutes for eIF4E to stimulate compartmentalized translation initiation of HIV-1 unspliced mRNA [20]. Groom *et al.* [21], by using an *in vitro* transcription/translation assay from rabbit reticulocyte lysates, have demonstrated that Rev, which interacts with DDX3, stimulates translation of HIV-1 mRNAs at low concentration. This stimulation is dependent on a Rev binding site, in addition to the RRE, present in the internal loop B of stem-loop 1 (SL1) of HIV-1 RNA packaging signal [22]. On the contrary, at a high concentration, Rev inhibits mRNA translation in a non-specific manner. In this process, Rev may bind mRNAs and block ribosomal scanning by a mechanism that remains to be elucidated [21]. Lai* et al.* [23] have shown that DDX3 is recruited to the TAR region through the interaction with the Tat protein in order to promote HIV-1 mRNA translation.

Other cellular proteins also bind the TAR element and promote viral translation. RNA Helicase A (RHA) binds to the TAR region* in vitro* and* in vivo* enhancing HIV-1 LTR-directed gene expression and viral production [24]. Dorin* et al.* [25] showed that the TAR RNA-binding Protein (TRBP) promotes translation of TAR-bearing RNAs independently of its ability to inhibit protein kinase RNA-activated (PKR). Other RNA-binding proteins have been shown to stimulate HIV-1 translation. This is the case with Staufen [26] and La autoantigen [27]. Both proteins stimulate translation of TAR-containing reporter transcripts.

### 3.2. The Vpu-Env Bicistronic mRNA: Modulation of Ribosome Scanning Process

**Figure 3:**
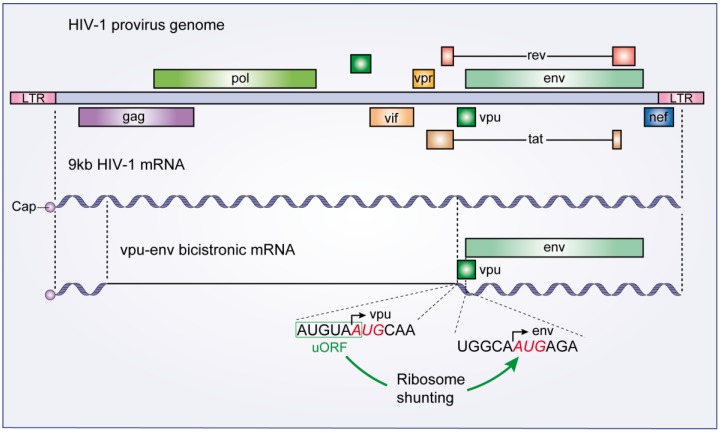
The HIV-1 vpu-env bicistronic mRNA. A schematic diagram of the HIV-1 provirus genome is presented along with its primary 9 kb mRNA transcript. The vpu-env bicistronic mRNA is formed after splicing of the 9 kb mRNA. Start codon sequences of both vpu and env ORFs are presented and the upstream ORF that stimulates Env translation is boxed in green.

As many other viruses, HIV-1 optimizes its genome coding capacity by translating different proteins from a common mRNA. This is the case of the bicistronic vpu-env mRNA which encodes both Vpu and Env proteins [28,29]. The coding sequences of Vpu and Env proteins are arranged so that the Vpu Open Reading Frame (ORF) precedes the Env coding sequence (Figure 3). Schwartz* et al.* [28,29] showed that the Env protein is synthesized by a leaky scanning mechanism in which the 43S PIC passes through the Vpu start codon. According to these authors, this modulation of the 43S PIC scanning process is achieved because the Vpu initiation codon is surrounded by a weak Kozak context [28,29]. As a result, mutations of the Vpu initiation codon that improve its Kozak context inhibit Env translation from the bicistronic vpu-env mRNA [28,29].

In contrast, by studying the 5'UTR of 16 alternatively spliced Env mRNAs, some of which also including the extra upstream Rev initiation codon, Anderson* et al.* [30] demonstrated that mutations in the upstream AUG codons of the Env ORF had little effect on Env synthesis. This suggests that Env translation is achieved via a discontinuous scanning mechanism such as ribosome shunting, a process in which the ribosome bypasses parts of the 5'UTR to reach a start codon. Indeed, Krummheuer* et al.* [31] reported that translation of Env protein was inconsistent with the leaky scanning model. Instead, Env translation is stimulated by a six-nucleotide upstream ORF (uORF) (Figure 3), which is located in the vpu start codon region. uORFs are short open reading frames located within the 5'UTR of a mRNA. Mutations of the start and stop codons of this uORF reduced Env protein translation five-fold [31]. The authors suggest that this uORF acts as a ribosome pausing site supporting the ribosome shunting model [31].

### 3.3. The Fate of Unspliced HIV-1 mRNA

#### 3.3.1. Cap- and IRES-Dependent Translation Initiation

The HIV-1 unspliced mRNA can initiate translation of Gag and Gag-Pol polyproteins either by a classical cap-dependent mechanism or by Internal Ribosome Entry Sites (IRES) [32,33,34,35]. Cap-dependent translation of HIV-1 unspliced mRNA has been observed* in vitro* [35] and* ex vivo* [33,36]. As described above, cap-dependent translation initiation relies on the recognition of the 5'UTR cap structure by the eIF4F complex to promote the recruitment of 43S PIC (Figure 1).

**Figure 4:**
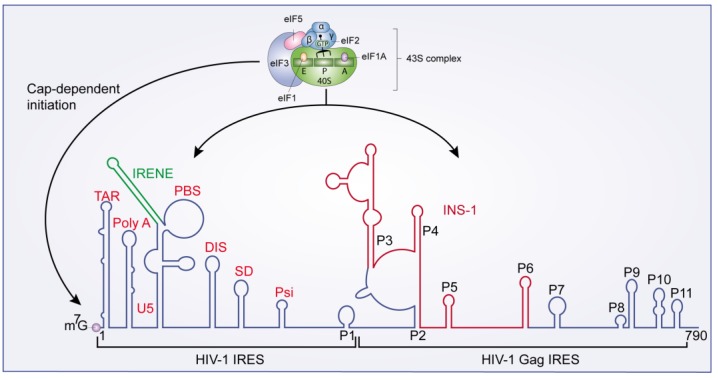
HIV-1 IRESs. HIV-1 IRES and HIV-1 Gag IRESs are represented. The 43S pre-initiation complex can mediate both cap-dependent and IRES-mediated translation initiation.

IRESs present in several viral mRNAs (mainly picornaviruses and Hepatitis C virus) overcome a global translation down-regulation established by the cell during viral infection. The HIV-1 genomic mRNA presents two IRESs: one in the viral 5'UTR, named HIV-1 IRES [32] and a second within the *gag* coding region, known as HIV-1 Gag IRES [37] (Figure 4). The HIV-1 IRES has been characterized in a proviral wild-type HIV-1 clone (pNL4.3) [32], but also in the CXCR4 (X4)-tropic primary isolate HIV-LAI [38], and in viral RNA isolated from clinical samples [39], demonstrating the importance of these elements in HIV-1 replication. The cap structure and the HIV-1 IRES both drive translation of pr55^Gag^ and pr160^Gag/P^°^l^. The minimal active HIV-1 IRES is harbored within the region spanning nucleotides 104 to 336 [32]. This region also contains several RNA motifs involved in different functions of the HIV-1 life cycle (Figure 4).

The HIV-1 IRES has been shown to be implicated in different cellular states in which cap-dependent translation initiation is inhibited [18]. Monette* et al.* [34], by using* in vitro* artificial systems and HIV-1-expressing cells, reported that Pr55^Gag^ translation was preserved at 70% when eIF4G and PABP, two main components of the cap-dependent translation initiation, were targeted by picornavirus proteases. Using a similar system, Amorim *et al.* [40] showed that HIV-1 protein synthesis is highly dependent on cap-initiation the first 24–48 h of viral replication, while at later time points IRES-dependent translation is needed to ensure viral particles production. HIV-1 IRES also drives viral structural protein synthesis during the G2/M cell cycle transition [32,41] and is stimulated by oxidative stress [38].

Although the molecular mechanism of the HIV-1 IRES-mediated translation remains largely unknown, Plank* et al.* [42] demonstrated that the HIV-1 IRES is cell type-specific. Using a plasmid encoding a dual-luciferase reporter mRNA, the authors showed that the HIV-1 IRES activity was 4-fold higher in Jurkat T-cells than in HeLa cells. Based on the fact that all spliced and unspliced HIV-1 transcripts possess the same 289 nt long 5'UTR, the authors also demonstrated that vif, vpr, vpu, and nef transcripts can initiate translation by an IRES. Interestingly, despite the fact that all HIV-1 constructs contain the same 5' leader region, the IRES activity of these transcripts differ from the IRES-containing gag transcript [42]. Based on these results, Plank* et al.* [42] proposed a model in which the structure of the 5' leader region adopts several conformations to stimulate different processes in the viral replication cycle, such as RNA dimerization prior to packaging or IRES-mediated translation. Thus, cell type-specific ITAFs (IRES Trans-Acting Factors) could promote the structural conformation of the 5' leader region required for an optimal IRES activity. In vif, vpr, vpu or nef transcripts, the specific ITAFs bind regions of the 5'UTR common to all these transcripts to form a “Core IRES” that stimulate IRES-mediated translation. However, the IRES activity of these transcripts could be regulated by specific RNA sequences present downstream of the common 5'UTR [42].

Several cellular proteins have been reported as cellular factors increasing the HIV-1 IRES activity [18]: eIF5 [43], the heterogeneous nuclear ribonucleoprotein A1 (hnRNP A1) [44] and the Rev-cofactors DDX3 and hRIP [43]. On the contrary, the human embryonic lethal abnormal vision (ELAV)-like protein (HuR) has been identified as a negative regulator of the HIV-1 IRES activity [45]. *Cis*-acting elements have also been shown to negatively modulate HIV-1 IRES activity. Brasey *et al.* [32] showed that the Gag ORF impacts the HIV-1 IRES-mediated translation initiation in the context of a bi-cistronic mRNA. Gendron* et al.* [38] identified another region located upstream of the PBS, the IRES negative element (IRENE) that also negatively regulates the HIV-1 IRES activity (Figure 4). Recently, it was shown that the instability element 1 (INS-1), a *cis*-acting regulatory element present within the gag ORF, also inhibits HIV-1 IRES activity (Figure 4) [46]. Furthermore, several HIV-1 IRES ITAFs have been identified from G2/M-arrested cell extracts [41], including proteins that have already been identified to have a role in HIV-1 replication, such as the high mobility group protein HMG-I/HMG-Y (HMG-I(Y)) [47] or the activated RNA polymerase II transcriptional co-activator p15 [48].

The HIV-1 Gag IRES mediates translation of both full length Pr55^Gag^ polyprotein and a *N*-truncated 40-kDa Gag (p40) isoform lacking the matrix domain [37,49]. Despite the fact that p40 is produced at a much lower level than Pr55^Gag^, this protein seems to be important in wild-type replication of HIV-1 in cultured cells [37]. Expression of p40 has also been observed in all HIV-1 clinical isolates from a large cohort of patients (*n* = 100) [18].

#### 3.3.2. Ribosomal Frameshift

**Figure 5:**
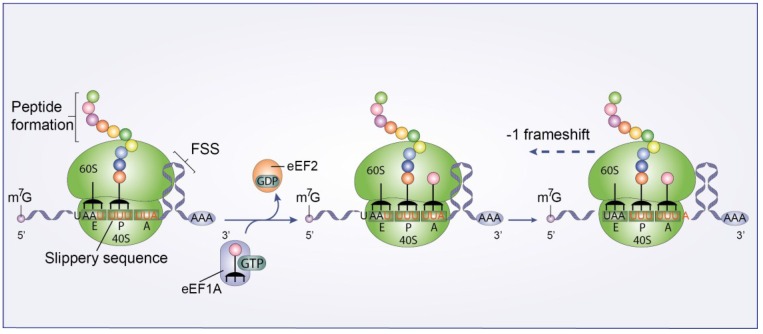
HIV-1 Ribosomal frameshift. A schematic diagram of the −1 nucleotide ribosomal frameshift. This process is mediated by a slippery heptanucleotide sequence (UUUUUUA) where the frameshift takes place, and an RNA element called the frameshift stimulatory signal (FSS).

The Gag-Pol polyprotein, which is synthesized at a ~1/10 ratio compared to Gag, is translated via a −1 nucleotide ribosomal frameshift [50,51]. This process is regulated by two main factors, a slippery heptanucleotide sequence (UUUUUUA) where the frameshift takes place, and a downstream RNA element called the frameshift stimulatory signal (FSS) that controls the frameshift efficiency (Figure 5) [52]. The shift places the *gag* termination codon into an out-of-frame context and translation continues toward the downstream *pol* sequence [50,51]. The structure of the FSS and its mechanistic mode of action are not well understood. It has recently been proposed that the −1 frameshift is promoted by 4 pseudoknots (PK1-4) present in the FSS [53,54]. However, *ex virio* SHAPE (Selective 2'-Hydroxyl Acylation analyzed by Primer Extension) experiments [55] and frameshifting assays [56] do not support this model.

The most widely accepted model suggests that −1 frameshift is triggered when the FSS forces a small portion of the 80S complexes to make a pause. This pause forces the 80S complexes to shift one nucleotide backwards into the *pol* sequence [57,58,59]. Léger* et al.* [57] have proposed a model in which, after release of the eEF2-GDP complex from the ribosome, the two tRNAs cannot be translocated by three nucleotides (from P/A to E/P sites). Instead the two tRNAs are translocated only by two nucleotides due to the FSS. Consequently, the two tRNAs are trapped in an intermediate translocation state and frameshift is achieved [57]. This is supported by the cryo-EM structure resolved by Namy* et al.* [58], in which the ribosome is stalled by the presence of the FSS. Additionally, Mouzakis* et al.* [56] have demonstrated that the base pairs important for this process are located at an 8 nt distance from the slippery sequence, which is consistent with the paused-ribosome model.

Further studies have shown that the highly structured TAR element and the Tat protein modulate ribosomal frameshift [60,61]. Charbonneau* et al.* [61] demonstrated that the presence of the HIV-1 5'UTR in a reporter mRNA increases the −1 frameshift efficiency fourfold in Jurkat T-cells, compared to a control reporter with a short unstructured 5'UTR. This is associated with the presence of the TAR region within the 5'UTR [60,61]. This region slows down the rate of translation initiation during cap-dependent translation. As a consequence, the distance between ribosomes is larger and the FSS has more time to refold [60,61]. Thus, a structured FSS is required for an optimal −1 frameshift process. The increase in the −1 frameshift efficiency is antagonized by the HIV-1 Tat protein, which indirectly destabilizes TAR structure by increasing recruitment of RNA helicases, such as DDX3 [23]. Moreover, Lorgeoux* et al.* [62], using a dual-luciferase reporter assay, showed that the helicase DDX17 is required for −1 frameshift and for maintaining proper ratios of Gag* vs.* Gag-Pol proteins.

### 3.4. Redirecting mRNA Activation

In the G2/M phase of the cell cycle, cap-dependent translation is down-regulated by a cascade of events that lead to the disruption of the eIF4F complex, and thereby inhibition of the mRNA activation step (Figure 1, step 3) [63]. In this process, formation of the eIF4F complex is prevented by targeting of eIF4E. This factor is regulated by a family of translation inhibitor proteins, named the eIF4E-binding proteins (4E-BPs). After hypophosphorylation, 4E-BPs compete with eIF4G for the same binding site on eIF4E, preventing eIF4F complex assembly [63,64].

In HIV-1-infected cells, viral proteins alter cell function by affecting different cellular pathways. HIV-1 viral protein R (Vpr) has been identified as a viral protein capable of arresting cells in the G2/M phase. Vpr mediates G2/M arrest via a complex signaling cascade involving activation of the ataxia telangiectasia mutated and Rad3-related kinase (ATR) [65]. Vpr-induced G2/M arrest also inhibits host protein translation by a process involving the regulation of eIF4E activity (Figure 1C) [66].

Despite the global reduction of host protein synthesis by Vpr-induced G2/M arrest, translation of HIV-1 structural proteins is maintained [58]. RNA-coimmunoprecipitation experiments showed that full-length unspliced HIV-1 genomic RNA and singly spliced mRNAs are associated with CBC in contrast to multi-spliced viral and cellular mRNAs that are associated with eIF4E. Moreover, unspliced and singly-spliced viral mRNAs retain their interaction with CBC during translation and packaging. Based on these observations, Sharma* et al.* [66] hypothesized that unspliced and singly-spliced viral mRNAs are translated due to the ability of the CBC to activate mRNA during initiation (Figure 1A). Thus, CBC retention could allow viral protein synthesis while the global protein translation is inhibited by an eIF4E decrease.

### 3.5. Targeting Cellular Translation Factors

During the HIV-1 replication cycle, viral proteins inhibit different translation factors. This is the case of the HIV-1 protease, which partially impairs cap-dependent protein translation, in addition to its main function during virion maturation. Ventoso* et al.* [67] were the first to report that HIV-1 protease is able to cleave the initiation factor eIF4G *in cellula* (Figure 1B), by using HIV-1 C8166 target cells. Further studies using rabbit reticulocyte lysates have demonstrated that HIV-1 protease not only cleaves eIF4G but also PABP (Figure 1B) [68,69,70]. Degradation of eIF4G and PABP leads to an inhibition of the cap- and PABP-dependent translation initiation [67,68,69,70]. Jäger* et al.* [71] reported that the HIV-1 protease also cleaves eIF3d (Figure 1B), a subunit of eIF3. This cleavage presents a similar efficiency to the one of the Pr55^Gag^. This may also promote inhibition of cap-dependent protein synthesis.

In the early phases of HIV-1 replication, the HIV-1 TAR RNA element activates PKR, which mediates host translation inhibition [72]. PKR interacts with the TAR element, inducing PKR dimerization, auto-phosphorylation and activation. Activated PKR phosphorylates the alpha subunit of eIF2 (eIF2α), blocking its recycling for ongoing translation, resulting in a potent translation inhibition of cellular and viral mRNA [73]. HIV-1 indirectly prevents phosphorylation by targeting PKR. Indeed, the HIV-1 Tat protein directly interacts with PKR, thus, preventing auto-phosphorylation (Fig 1D) which is essential for function [72,74]. Moreover, during the late events of HIV-1 replication, PKR activity is inhibited by the high concentration of the HIV-1 TAR element (Fig 1D). Studies with TAR and other RNAs showed that high concentration of double-stranded RNA (dsRNA) inhibits PKR dimerization and therefore its activation [75]. PKR activation is further inhibited by HIV-induced mechanisms involving the cellular factors TRBP, Adenosine deaminase ADAR1 and a change in PACT function [72,76].

Moreover, by using a yeast two-hybrid screen assay, Cimarelli and Luban [77] reported that the HIV-1 Pr55^Gag^ binds to eEF1A through its matrix (MA) and nucleocapsid (NC) domains. They demonstrated that this interaction requires RNA, and suggested that tRNA could probably mediate this interaction since both NC and eEF1A have been shown to bind tRNAs [78,79]. Thus, as cellular concentration of Pr55^Gag^ increases in the cell, Pr55^Gag^ association with eEF1A-tRNA complexes may induce translational inhibition (Figure 2A). As a consequence of this inhibition, viral genomic RNA could be released from the translation machinery. Subsequently, Pr55^Gag^ interaction with the genomic RNA may lead to a further viral translation inhibition, thus stimulating RNA packaging into virions [77]. The authors also showed that both eEF1A and a truncated form of eEF1A of 34 to 36 kDa are incorporated into virions. In addition, eEF1A can also interact with the cellular cytoskeleton, suggesting a possible role of eEF1A in virion assembly and budding [80]. Despite these reports, direct evidence for a role of eEF1A in RNA packaging, virion assembly or viral particle budding remains to be demonstrated [81].

Similar to Pr55^Gag^, HIV-1 Nef protein also interacts with eEF1A and forms a Nef/eEF1A/tRNA complex. Thus, Nef mediates a nucleocytoplasmic relocalization of eEF1A and tRNAs to prevent stress-induced apoptosis in primary human macrophages (Figure 2B) [82]. Moreover, Warren* et al.* [83] have reported that the HIV-1 reverse transcription complex (RTC) recruits eEF1 to stimulate late steps of the HIV-1 reverse transcription process (Figure 2C). The eEF1 factor binds to the HIV-1 RTC through an interaction with reverse transcriptase (RT) and integrase (IN). This association enhances the stability of the RTC in the cytoplasm [83]. Nevertheless, further studies will be needed to establish whether eEF1 functions synergistically with the components of the RTC, or independently during reverse transcription.

### 3.6. Inhibiting APOBEC3G Translation

APOBEC3G (*apo*lipoprotein *B* mRNA-editing enzyme, *c*atalytic polypeptide-like *3G,* or A3G) is a restriction factor that impairs HIV-1 replication [84]. A3G is incorporated into viral particles and during reverse transcription in the target cells, it converts cytidines into uridines in the (−) strand DNA. This will generate further reverse transcription and integration defects and potentially produce non-functional viral proteins. HIV-1 counteracts this cellular factor by at least two pathways involving the HIV-1 viral infectivity factor (Vif). First, binding of Vif to A3G allows the recruitment of an E3 ubiquitin ligase that mediates the poly-ubiquitination of A3G and its degradation through the proteasome pathway. Second, Vif impairs the translation of A3G mRNA through a putative mRNA-binding mechanism [85,86].

Mariani* et al.* [87] were the first to observe that Vif inhibits A3G translation and suggested that this repression may contribute to the reduction of A3G encapsidation. The authors reported that Vif causes a 4.6 fold reduction in A3G synthesis by pulse-chase metabolic labeling experiments. Additionally, Kao* et al.* [88] showed that Vif causes the reduction of cell-associated A3G by 20%–30% compared to up to 50-fold reduction in virus-associated protein. This result supports the idea that Vif functions at different levels to reduce the intracellular levels of A3G. A3G translational inhibition was finally confirmed by Stopak* et al.* [89]. By using kinetic analyses and* in vitro* transcription-translation experiments, the authors showed that Vif was capable of impairing A3G translation by approximately 30%–40% [89].

Based on these observations, we recently showed that Vif binds to A3G mRNA and inhibits its translation* in vitro* [86]. Indeed, filter binding assays and fluorescence titration experiments revealed that Vif tightly binds A3G mRNA. We also demonstrated that Vif is able to inhibit A3G translation in* in vitro*-coupled transcription/translation assays. In these experiments, Vif caused a two-fold reduction of A3G translation in a 5'UTR-dependent manner, most likely through mRNA binding and/or through its RNA chaperone activity [90,91]. These observations show that HIV-1 not only targets the host translational machinery to improve translation of its own proteins, but also inhibits translation of the host restriction factor A3G.

## 4. Concluding Remarks

HIV-1 has evolved several mechanisms that control the host translation machinery and overcome different obstacles in the cell, such as ribosomal scanning inhibition due to highly structured RNA elements and cap-dependent translation inhibition triggered by host immune responses. During this antiviral state triggered by the cell, HIV-1 proteins interact with the eukaryotic translation apparatus in different ways. Thus, these mechanisms not only promote translation of HIV-1 proteins, but also guarantee the fitness of newly produced viral particles by inhibiting A3G incorporation. It seems that the sophisticated functions presented in this review only represent a fraction of the strategies that have evolved by HIV-1 interaction with cellular functions, leading to a control of the host translation machinery during viral replication. Indeed, Jäger* et al.* [71] reported 497 interactions between 16 HIV-1 proteins and 435 human proteins. In addition, genome-wide techniques such as ribosome profiling [92] and/or iCLIP (individual-nucleotide resolution UV cross-linking and immunoprecipitation) [93] could be used, for example to identify HIV-1 Vif mRNA targets and to understand how this protein inhibits translation of specific genes. Identifying which cellular proteins of the translational eukaryotic machinery are targeted by HIV-1 will be crucial for a global understanding of the HIV-1 replication.
